# Modelling Transmission of Vector-Borne Pathogens Shows Complex Dynamics When Vector Feeding Sites Are Limited

**DOI:** 10.1371/journal.pone.0036730

**Published:** 2012-05-08

**Authors:** Arik Kershenbaum, Lewi Stone, Richard S. Ostfeld, Leon Blaustein

**Affiliations:** 1 Department of Evolutionary and Environmental Biology, and the Institute of Evolution, Faculty of Natural Sciences, University of Haifa, Haifa, Israel; 2 Department of Zoology, Tel Aviv University, Tel Aviv, Israel; 3 Cary Institute of Ecosystem Studies, Millbrook, New York, United States of America; Massey University, New Zealand

## Abstract

The relationship between species richness and the prevalence of vector-borne disease has been widely studied with a range of outcomes. Increasing the number of host species for a pathogen may decrease infection prevalence (dilution effect), increase it (amplification), or have no effect. We derive a general model, and a specific implementation, which show that when the number of vector feeding sites on each host is limiting, the effects on pathogen dynamics of host population size are more complex than previously thought. The model examines vector-borne disease in the presence of different host species that are either competent or incompetent (i.e. that cannot transmit the pathogen to vectors) as reservoirs for the pathogen. With a single host species present, the basic reproduction ratio *R_0_* is a non-monotonic function of the population size of host individuals (*H*), i.e. a value 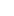 exists that maximises *R_0_*. Surprisingly, if 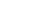 a reduction in host population size may actually increase *R_0_*. Extending this model to a two-host species system, incompetent individuals from the second host species can alter the value of 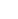 which may reverse the effect on pathogen prevalence of host population reduction. We argue that when vector-feeding sites on hosts are limiting, the net effect of increasing host diversity might not be correctly predicted using simple frequency-dependent epidemiological models.

## Introduction

Zoonotic diseases show complex dynamics that are influenced by a wide range of ecological factors. Understanding these influences is important for the design of disease control strategies, because the outcome of ecological interventions may not always be intuitive [1], [2]. Much attention has been given to the effect of biodiversity on zoonotic disease spread, and in particular to the effect of alternative host species on the dynamics of vector-borne diseases. The term “dilution effect” (*sensu* [3]–[5]) describes the reduction in infection prevalence when a vector can feed on more than one host species. Hosts vary in their competence as pathogen reservoirs, generally with one or a few species being efficient (competent hosts) and others being inefficient reservoirs (incompetent hosts) [6]. From the perspective of the pathogen, bites on incompetent hosts are “wasted”, in that they cannot result in transmission. Therefore, presenting a vector with the opportunity to feed on an additional host that is less competent at pathogen transmission will result in less pathogen transmission. The reduction in pathogen transmission in the presence of an incompetent host species is a separate effect from the reduced transmission observed in a single-host system when pathogen prevalence is low, as the dilution effect alters the dynamic equations of the host-pathogen system. Mathematical models predict that the dilution effect would be expected to operate under a wide range of conditions [4], [7]–[10]. Some empirical studies have supported these predictions (e.g. [11]–[13]), while other studies have shown that increasing host species richness can have mixed effects (e.g. [14]).

**Figure 1:**
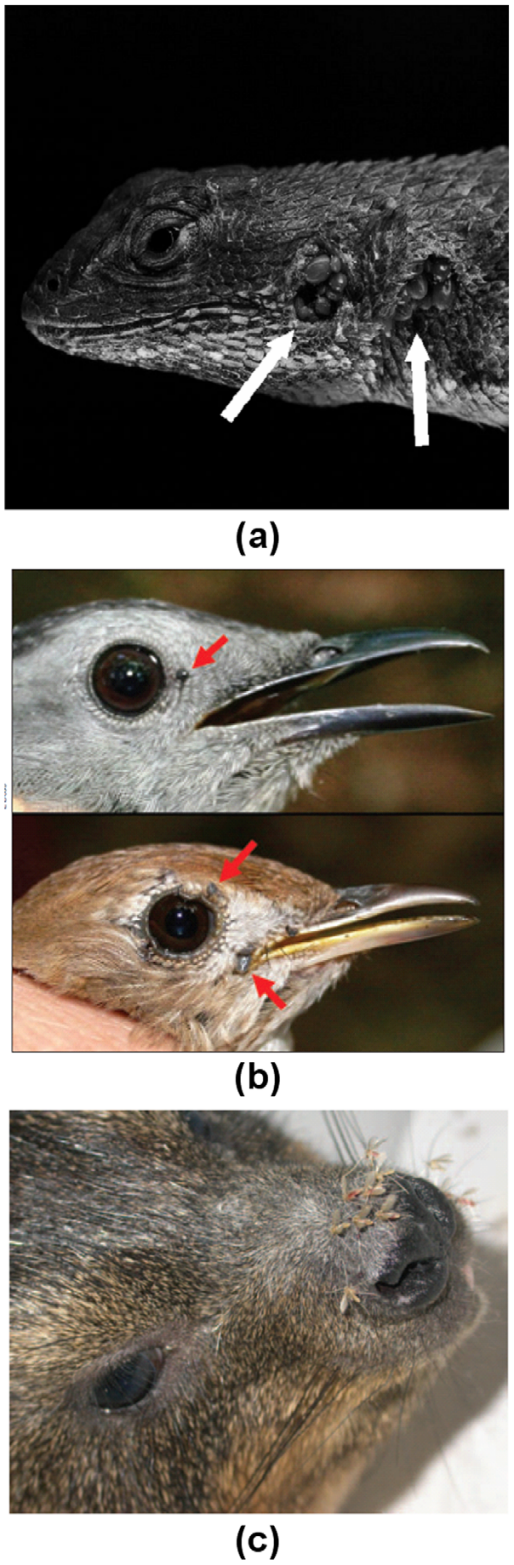
Examples of ectoparasites restricted to limited feeding sites on different species. Panel (a) shows western blacklegged ticks, *Ixodes pacificus* (indicated with arrows), restricted to two sites on the head of the western fence lizard, *Sceloporus occidentalis* (from Swei et al. [21] *Reproduced with the author’s permission*). Panel (b) shows ticks (indicated with arrows) restricted to the featherless areas of a gray catbird *Dumetella carolinensis*, and a hermit thrush *Catharus guttatus* (from Brinkerhoff et al. [22] *Reproduced with permission of the Ecological Society of America*). Panel (c) shows sandflies *Phlebotomus spp.* congregating on the furless snout of the rock hyrax *Procavia capensis* (from Svobodova et al. [23] *Courtesy of the Centers for Disease Control and Prevention*).

We develop a model in which vector biting is limited by a finite number of feeding sites on each host. If the host is large, the vector may never reach densities where feeding sites are limiting (e.g. horses and horseflies, [15]). However, when the host is small and has little exposed skin (e.g. snout and ear pinnae in mammals, or conjunctiva in birds), or when the host can use effective grooming behaviour over most of its body, the number of vector individuals able to feed at the same time is limited [16]. A limited number of feeding sites is probably the case, for example, with ticks (removed from most parts of the body by grooming) and sandflies (small proboscis and delicate body prevents penetration of thick hair) [17], [18]). Abundant observational evidence exists (e.g. [19]) for the hypothesis that parasite feeding success is regulated by the number of available feeding sites in certain species. Tyre et al [20] suggest that limited feeding sites may explain density-dependent engorgement success of the tick *Aponomma hydrosauri* on the sleepy lizard *Tiliqua rugosa*. Figure 1 shows three examples of hosts where parasite attachment is limited to specific body parts. The photograph from Swei et al. [21] (Figure 1a) demonstrates the restriction of western blacklegged ticks, *Ixodes pacificus*, to two scale-free sites on the head of the western fence lizard, *Sceloporus occidentalis*. The photograph from Brinkerhoff et al. [22] (Figure 1b) shows ticks attaching to the featherless areas of a gray catbird (*Dumetella carolinensis*) and a hermit thrush (*Catharus guttatus*), around the eyes and beak. The photograph from Svobodova et al. [23] (Figure 1c) shows sandflies (*Phlebotomus* spp.) congregating on the furless snout of the rock hyrax (*Procavia capensis*). Hawlena et al [24] found a low variance in flea engorgement on rodents at high flea density, implying parasite intraspecific competition for feeding site acquisition. However, quantitative estimates of the effect of limited feeding sites have not been made. Such conditions lead to a complex functional response of host-vector mixing to host-vector abundance, which must be modelled differently from traditional epidemiological models.

After having defined our model, we will derive expressions for the initial basic reproduction ratio (*R_0_*), which estimates the average number of secondary infections in competent hosts produced by a typical infected individual in a wholly susceptible population [25]. The classical theory predicts that if *R_0_*>1, the pathogen can invade and persist, but when *R_0_*<1 the pathogen will die out. We examine the dependence of *R_0_* on the relative numbers of two different species of pathogen hosts. We ask under what conditions of relative population sizes of both competent and incompetent host species the disease would be expected to become either enzootic or extinct. We then derive a specific implementation of this general model, and use the dynamic equations to predict the relationship between *R_0_* and the equilibrium prevalence.

## Methods

We present two models. The first is based on a discontinuous transition from feeding site saturation to excess feeding sites (Simple Discontinuous Model). In this model, we assume that vectors fill up feeding sites on the hosts whenever they are available. In the second model, we relax this assumption and allow feeding success probability to vary continuously with the number of feeding sites available, making vector feeding success less likely as more feeding sites become occupied (Vector Interference Model).

We consider constant population sizes (competent hosts, *H*; incompetent hosts, *M;* and vectors *V*), i.e. each individual that dies is replaced by a new individual. We assume that vector population size is not dependent on host population size. Although few comprehensive reviews of this assumption exist [26], it is supported by studies of specific species and is accepted by many researchers because haematophagous arthropod reproduction may be limited by the availability of breeding sites, rather than by blood meals [26]. Preliminary investigations showed that our results appear to be robust to relaxation of this assumption. Regarding host population size, the detrimental effect of vector feeding on host fitness may not limit host reproduction [27], [28]. For these reasons, we choose to consider both host and vector populations as constant over time, ignoring any transient changes in population size. By holding population sizes constant, we can show that dilution/amplification can occur as the result of biodiversity changes, independent of population size effects.

A further assumption is that a vector always finds a feeding site on a host, if a site is available. Little data exist on vector mortality while searching for hosts [29], but this may be a realistic assumption in many systems. Spatial effects of non-uniformly distributed individuals, non-overlapping populations, or spatially limited searching are not considered.

We first analyse the system around the infection-free state in order to derive expressions for *R_0_* when the pathogen prevalence is low. We then show that this can be extended to a dynamical model without the assumption of infection rarity, and use this to predict equilibrium prevalence.

## Results

### Simple Discontinuous Model

We consider that each host has a limited number of feeding sites, which is on average, *k*. The probability of a host being fed upon differs among species, since the two host types *H* and *M* have a different average number of feeding sites available: *k_h_* and *k_m_* respectively. Therefore, from the perspective of the vector, there are a total of *N* feeding sites available in the host population where:
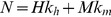
(1)Clearly, the system can operate in one of two modes: (a) where there are insufficient feeding sites for all vector individuals, *V>N*, and (b) where there are enough feeding sites for all vector individuals, *V<N*. In mode (a) only some vector individuals feed, and in mode (b), all vector individuals take a blood meal. Initially, we take each of these cases separately, and deal independently with these (a) saturated, and (b) unsaturated cases, so that the model is discontinuous with respect to *(V,N)*. Later, we relax this assumption in the Vector Interference Model which uses a single continuous equation for all *(V,N)*. To determine *R_0_* for this model, reasonable parameter values were chosen, based on the assumption of a large competent host and a smaller incompetent one, but the qualitative predictions of the model apply in any case where the number of feeding sites on each host type is different (e.g. hosts of different sizes, thereby having different surface areas on which vectors can feed). A description of all the symbols used in our model is given in Table 1.

**Table 1:** Parameters used in the simulations.

Parameter	Meaning	Value
β	Probability of infection following contact	0.2
γ*_h_*	Clearance rate of host	0.4/day
γ*_v_*	Clearance rate of vector	0.5/day
*k_h_*	Number of feeding sites on competent host	30
*k_m_*	Number of feeding sites on incompetent host	3
*V*	Number of vectors	1000
ϕ	Density dependent feeding interference	*e* ^−1^

To derive the basic reproduction ratio *R_0_*, we calculate the number of new infected hosts per initial infected host, over the average infectivity time of that host in a fully susceptible host population. However, in the model, we assume a time step of the natural feeding cycle of the vector, and although it need not be explicitly defined, it will be shorter for mosquitoes and sandflies, and longer for ticks. We make no specific assumptions about the length of the feeding cycle, although for the purpose of parameterisation, we have chosen an arbitrary cycle length of one day. In calculating the number of newly infected vectors per infected host, we consider first the transmission from host to vector, and then from vector to host. In the saturated case (a), all feeding sites on all hosts are occupied and therefore each host is fed upon by on average *k_h_* vectors. In the unsaturated feeding site case (b), the *V* vectors are distributed among the *N* feeding sites. Each competent host has on average *k_h_* feeding sites, and therefore on average, *k_h_V/N* vectors. β is the transmission rate, and suppose the host infectious lifespan is *1/*γ*_h_*, where γ*_h_* is the infected host removal rate by death or recovery. We then have the following expressions (2) for the average number of newly infected vectors (Δ*V_I_*) for each infected host (*H_I_*) over the average host infectious lifespan.
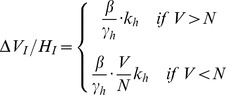
(2)The expected number of new infected hosts per infected vector can be similarly derived (4). In the saturated case, the probability of a vector finding a host of any species to feed upon is *Pr_feeding_ = N/V*, and the probability of this being a competent host as opposed to an incompetent one is *Pr_H_ = Hk_h_/N*. Therefore, the total probability of a vector biting a competent host is the product of these two terms: *Hk_h_/V*. Conversely, in the unsaturated case, the probability of finding a feeding site is unity *Pr_feeding_ = *1, and therefore the probability of biting a competent host is simply *Pr_H_* = *Hk_h_*/*N*.



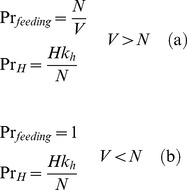
(3)Since we are considering the limiting case of the introduction of a single infected host into a system with no infected vectors, we can ignore the possibility that a susceptible host will be bitten by more than one infected vector given the rarity of such an event. The average number of new infected hosts (Δ*H_I_*) per infected vector (*V_I_*) (4) is given in a similar way to Equation (2):
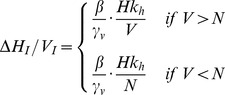
(4)


Here, γ*_v_* is the vector removal rate. The basic reproduction ratio *R_0_* can be calculated using its definition as the number of new cases per original case. The number of new infected hosts over the lifetime of the original infected host (5) is the product of the terms in (2) and (4).
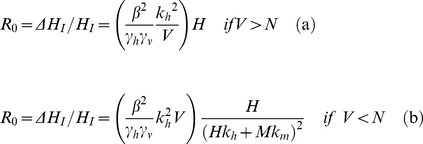
(5)


This expression can be simplified by substituting:
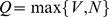
(6)which yields:



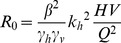
(7)The expression in (7) gives *R_0_* for the general case of a system of both competent and incompetent hosts. The expression represents *R_0_* as the number of newly infected hosts after a single cycle involving two transmission steps: host to vector and vector to host. This result is consistent with that obtained when *R_0_* is derived as the largest eigenvalue of the “next generation matrix” [30]. In this case, we can derive the Jacobian matrices separately for the appearance of new infections (*F*) and the loss of infective individuals (*V*). *R_0_* at the disease-free equilibrium is then given by the largest eigenvalue of *FV* ^−*1*^. The Jacobian matrices *F* and *V* at *(V_I_,H_I_) = (0,0)* are given by:
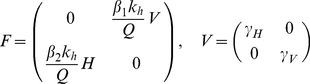
(8)


And the largest eigenvalue of *FV* ^−*1*^, and hence *R_0_*, is given by:
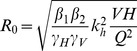
(9)


The square root that appears in Equation (9) is the result of the different interpretation of generation time in this analysis. Equation (9) expresses *R_0_* as the number of new host infections arising from a single host infection, after one generation of inter-species transmission; i.e. host to vector. Our Equation (7) provides a similar metric, but after one complete transmission cycle; host-vector-host. We prefer the use of the expression arising from Equation (7), without the square root, as it represents more intuitively the processes taking place, and so we make use of this expression in the analysis of equilibrium prevalence. Clearly, choosing one or other does not affect the location of the bifurcation where *R_0_ = √R_0_ = 1*.

Before considering the implications of this general system, we consider the case of a single (competent) host species *H* and a vector *V*. Without incompetent hosts (*M = 0*), the expression reduces to:
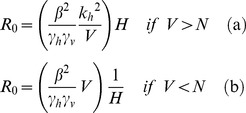
(10)


**Figure 2:**
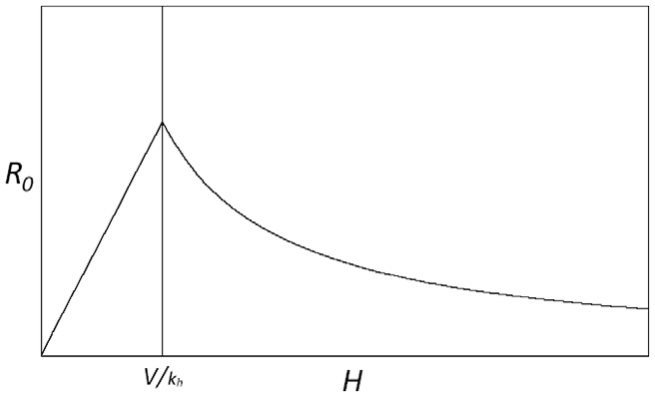
The response of *R_0_* to varying population size of host individuals (*H*) in a single-host system. Note that *R_0_* approaches zero for very small or very large values of *H*. The graph shows a discontinuity at the maximum level of *R_0_* at 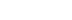

The expressions in (10) yield a surprising result: *R_0_* is proportional to *H* in the low *H* regime, but proportional to *1*/*H* at high *H*. At the boundary *V = N* the gradient of *R_0_* with respect to *H* changes from positive to negative, representing a fundamental shift in the response of the disease system to additional host individuals. Equation (10) leads us to expect the functional form shown in Figure 2.

Examination of Equation (1) shows that the maximum *R_0_* occurs at 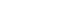 when there are exactly enough feeding sites for all the vector individuals. The non-monotonic dependence of *R_0_* on *H* could have critical importance for the control of disease via the manipulation of host species populations. As long as 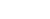 any reduction in *H* will lead to a reduction in *R_0_* and therefore potentially to a reduction in the disease prevalence. In epidemiological systems, attack rates are proportional to *R_0_* and equilibrium prevalence is positively correlated with *R_0_*, which we confirm for our system in a later section. However, if 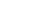 management strategies that attempt to reduce prevalence by reducing host numbers would have the undesired effect of increasing *R_0_* and therefore are likely to increase prevalence. The unrealistic discontinuity of the gradient at 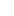
results from the simplifications present in this model, which we address later with the Vector Interference Model.

We now turn to the two host model, where *M>0*. From Equation (1), the boundary *H_b_* between the two regimes, *V>N* and *V<N* is given by:
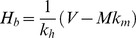
(11)


Since (5a) is independent of *M*, the dependence of *R_0_* on *H* in the saturated region remains identical to the single host species scenario. This is to be expected, since biting of competent hosts by vectors is at its maximum. There is no “waste” of bites that might otherwise transmit the pathogen, no matter how many incompetent hosts are present, because the feeding sites are saturated – every host is bitten by its full complement of vectors. We see this because *N* cancels when multiplying *Pr_feeding_* and *Pr_H_* in Equation (3). In the unsaturated regime, *V<N*, the expression for *R_0_(H,M)* is more complex (5b), and the functional form is determined by the relative values of *k_h_* and *k_m_* when *M* is fixed and *H* varied. This expression is, in general, non-monotonic, and may show a maximum *R_0_* for some value of *H>H_b_* in the unsaturated *V<N* regime. If the maximum of Equation (5b) occurs for *H<H_b_*, then *R_0_* is monotonically declining in the *V<N* regime. In order to determine which of these cases exists for particular values of parameters, we take the derivative of Equation (5b) with respect to *H*. The number of hosts *H* for which Equation (5b) is at a maximum can be shown to be:
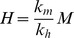
(12)


From (11) and (12), the condition for which this value of *H* gives the largest value of *R_0_*, and therefore 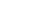 is:
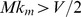
(13)


That is, if the incompetent host can provide feeding sites for at least half the vectors, then the maximum will occur in the unsaturated regime *V<N*. If condition (13) is not met, the maximum *R_0_* will occur at 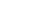 In summary:
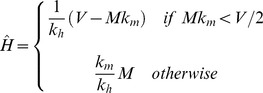
(14)


**Figure 3:**
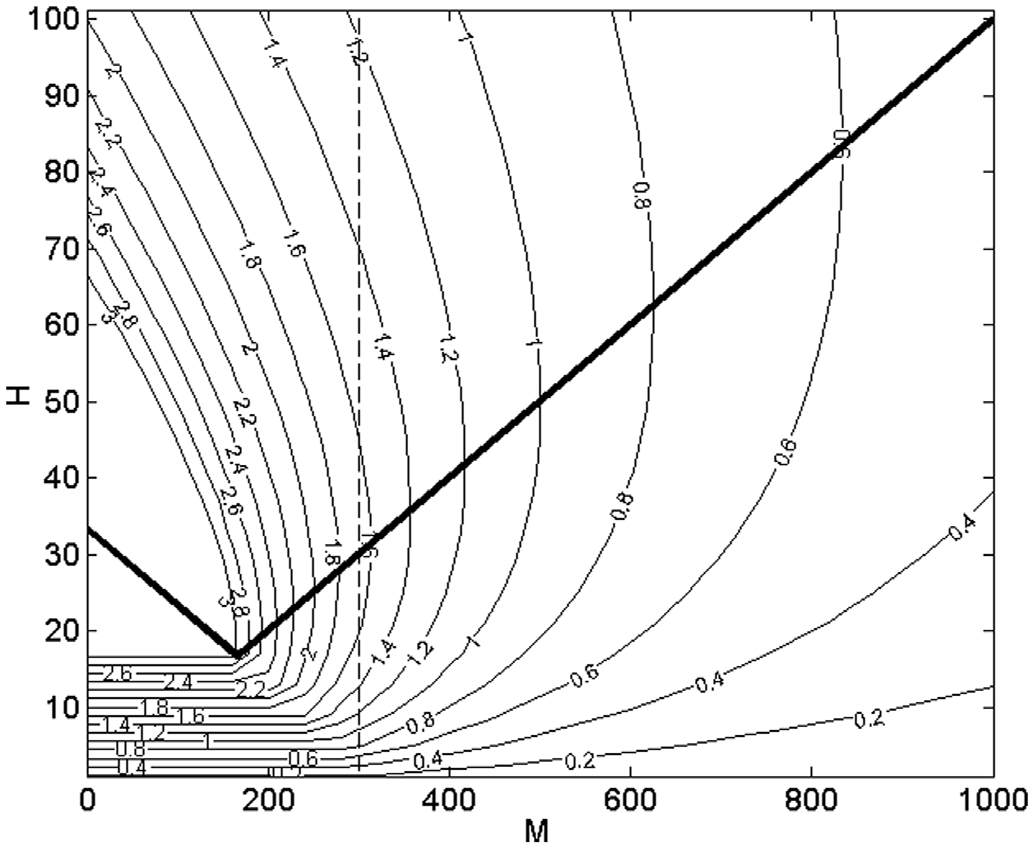
Contours of *R_0_* for varying population sizes of competent hosts (*H*) and incompetent hosts (*M*). The heavy line indicates the locus of maximum *R_0_* for any given *M*. Note that for a particular value of *M* (e.g. the dashed line) and low *H*, reducing the number of competent hosts has the effect of reducing *R_0_*. However, at higher populations of competent hosts, reducing the number of competent hosts will actually increase *R_0_*. Parameters used were as shown in Table 1.

If there was no saturation at all, and 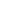 was determined solely by *Mk_m_/k_h_*, then in the absence of incompetent hosts, 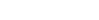 i.e. there would be only monotonic behaviour of *R_0_* in the single host system. The contours of constant *R_0_* are shown in Figure 3. It is instructive to note that the single host case (when *M = 0*) can be inspected in Figure 3 by examining the behaviour of *R_0_* along the vertical y-axis. Here the non-monotonic dependence of *R_0_* on *H* (Figure 2) can be seen as a specific case of the general behaviour in *H-M* space. The non-monotonic behaviour of *R_0_* (i.e. the presence of a maximum of *R_0_* for *M = 0*) exists because of the discontinuity at *V = N*, which causes the locus of maximum *R_0_* (thick line in Figure 3) to intersect the *H* axis at *H*>0. In the absence of the limiting effect of feeding sites, this locus would pass through *M = 0*, *H = 0*, and the non-monotonic effect seen in Figure 2 would not be observed.

**Figure 4:**
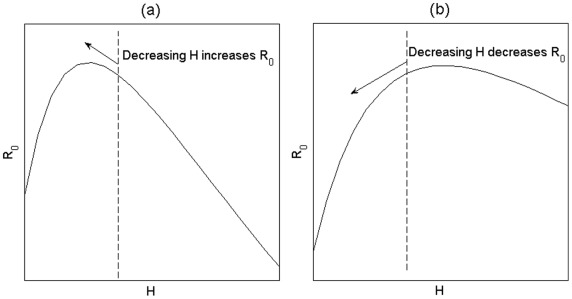
Non-monotonic response of *R_0_* at two different levels of *M*. (a) At low *M*, the level of *H* (dashed line) may be above that which gives the maximum *R_0_*. Therefore, decreasing *H* increases *R_0_*. (b) At higher *M*, when *H* is at or below the level that gives maximum *R_0_*, decreasing *H* decreases *R_0_*.

Non-monotonic behaviour does exist at values of *M>0*, and even in the unsaturated domain of *H-M* space, as shown in Figure 4. Increasing the number of incompetent hosts may cross the locus of maximum *R_0_*. In such a case, reducing host population numbers may either increase *R_0_* (Figure 4a) or decrease it (Figure 4b), depending on the (typically unknown) number of incompetent hosts present. In contrast to the example with a single host species, in this case the non-monotonic response of *R_0_(H)* is not the result of a saturated domain (*V>N*).

### Dynamic Model

The model described above in Equations 1–12 is a general abstract formulation for this form of vector-host system close to the infection-free state. We now show that the predictions of *R_0_* given in the previous section are preserved in a fully specified dynamical model. We use this to confirm the earlier prediction of the location of the transition between pathogen extinction (*R_0_*<1) and stable enzoonosis (*R_0_*>1), and to predict the equilibrium prevalence in the latter case.

We consider a basic SI compartment model [31], where hosts and vectors may be either Susceptible or Infected. Vectors do not recover from infection, but since every infected vector is eventually replaced by a susceptible vector individual (to satisfy the assumption of constant population size), the dynamic equations can be formulated as follows:
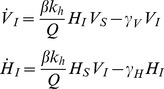
(15)where 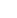 and 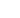 are the derivatives of *V_I_* and *H_I_* with respect to time. *Q* is the total number of vector bites: either *Q = V* or *Q = N* (where *N = Hk_h_+Mk_m_*) depending on whether the system is in the saturated or unsaturated regime (Equation 6). That is:
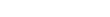



Since we choose to keep vector and host populations constant, the numbers of susceptible individuals are given by:
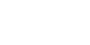
Which gives:



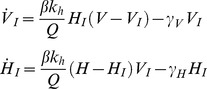
(16)Returning to Equation (16), we solve the differential equations for 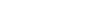 and find the fixed points (V_I_*,H_I_*), which represent an equilibrium solution. At equilibrium, P* = H_I_*/H represents the asymptotic disease prevalence – the proportion of hosts infected with the pathogen. Two solutions exist, (V_I_*,H_I_*) = (0,0), i.e. pathogen extinction, and a non-trivial enzootic solution:
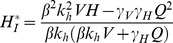
(17)The transition from *R_0_<1* to *R_0_>1* represents a transcritical bifurcation where the fixed point at *(0,0)* loses stability, and the enzootic fixed point becomes stable. The bifurcation can be located by examining the stability of the *(0,0)* fixed point, by linearising the system at the disease free equilibrium [32]. We calculate the Jacobian matrix of the system shown in Equation (16) for (V_I_*,H_I_*) = (0,0).
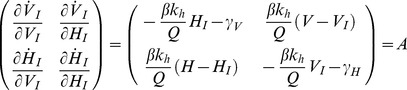
(18)given VI = 0, HI = 0
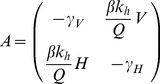
(19)Since the trace of this matrix, as shown by Equation (20), is always negative, the stability of the fixed point is determined by the determinant |*A*|.



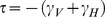
(20)If |*A*|>0, the fixed point (V_I_*,H_I_*) = (0,0) is stable and the pathogen becomes extinct. If |A|<0, then *(0,0)* is unstable and the enzootic solution shown in Equation (17) becomes stable. The transcritical bifurcation occurs at |*A*| = 0, hence:
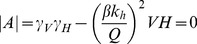
(21)and solving for *H*, we find an expression for *H_R0 = 1_* which represents the locus of *R_0_ = 1* for varying *M*. Recall that *Q = V* or *Q = N* depending on whether the system is in the saturated or unsaturated regime, so we obtain two alternative expressions for *H_R0 = 1_*



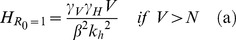
(22)

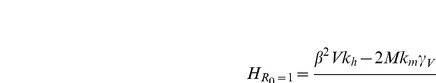



When *R_0_ = 1*, i.e. at the transcritical bifurcation, Equation (7) reduces to Equation (21) both for *V>N* (*Q = V*) and for *V<N* (*Q = N*). This is confirmation that the expression for *R_0_* derived in (7) is a reliable predictor of the ability of the pathogen to invade a disease-free system and become enzootic.

**Figure 5:**
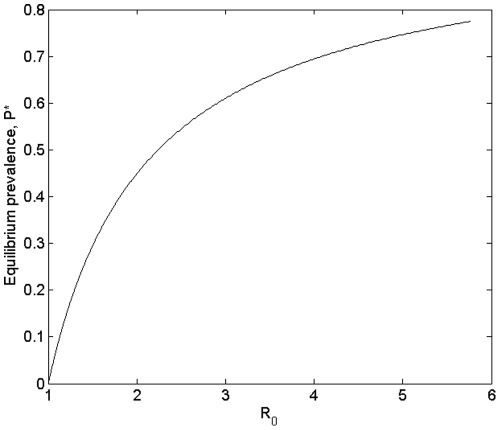
Equilibrium prevalence P* = H_I_*/H plotted against *R_0_*, as calculated for an arbitrary value of *H* = 32.

**Figure 6:**
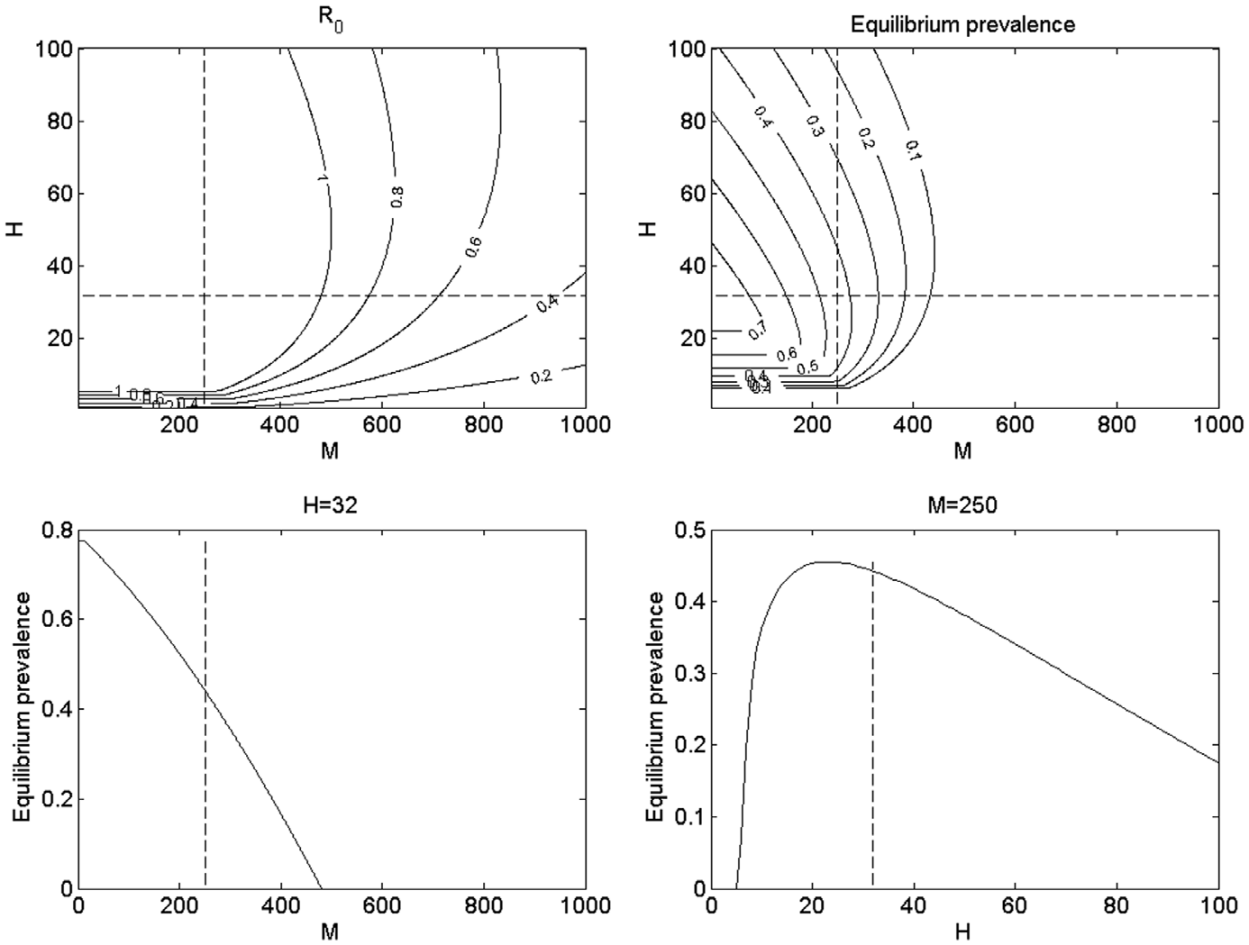
The response of equilibrium prevalence to varying *H* and *M*. Panel (a) shows contour lines of equal *R_0_* for varying *H* and *M*, as in Figure 3, and Panel (b) shows contour lines of equal equilibrium prevalence across the same parameter space. Dashed lines are shown for *H* = 32 and *M* = 250; Panel (c) shows the response of equilibrium prevalence as *M* is varied for *H* = 32 (dashed line indicates *M* = 250), and (d) shows equilibrium prevalence as *H* is varied for *M* = 250 (dashed line indicates *H* = 32).

We can illustrate the dependence of equilibrium prevalence on *R_0_* in the region of *R_0_*>1, by numerical evaluation of Equations (7) and (17), given that the equilibrium prevalence P* = H_I_*/H (Figure 5). This corroborates our previous claim that prevalence increases with increasing *R_0_*. Equilibrium prevalence follows a similar form to the response of *R_0_* across *H* and *M* parameter space, as can be seen in Figure 6a,b. Although for fixed *H* equilibrium prevalence declines with increasing M (Figure 6c), for fixed *M* prevalence shows a peak at some value of *H*, with a positive slope with respect to *H* at low values of *H*, but a negative slope at high values of *H* (Figure 6d); in a similar way to the response of *R_0_* shown in Figure 4.

### Vector Interference Model

Our characterisation of the system with a discontinuity at *V = N* is convenient, but probably unrealistic. Now we relax the assumption of vectors simply filling up available feeding sites on hosts. In reality, vectors will compete for feeding sites, and the probability of successful feeding will be reduced at higher vector densities through intraspecific competition [24]. In addition, host grooming and anti-parasite behaviour increases at higher vector loads [15], [33], [34], further decreasing the probability of an individual vector receiving a blood meal. We now incorporate a simplified representation of these effects into our model, and show that the essence of the dynamics is unchanged.

We assume that the probability of a vector receiving a blood meal is inversely related to the number of vectors per feeding site, according to the following relationship:
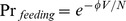
(23)where ϕ represents a measure of intraspecific feeding interference. Recalling from Equation (2) that the number of vectors attempting to feed from a host is *Vk_h_/N*, the number of vectors successfully feeding from a host is:



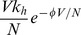
(24)It is convenient to set ϕ so that the maximum number of successful blood meals will be equal to *k_h_,* the number of feeding sites available. The maximum number of successful blood meals occurs when the number of vectors is given by
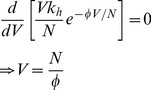
(25)


Since we have set the maximum number of successful blood meals to be *k_h_*, then:
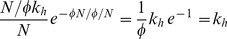
(26)


Therefore, we set ϕ* = e* ^−*1*^.

We rewrite Equation (7) as follows, for all *V*, remembering that at least two successful blood meals are required (one from host to vector and the other from vector to host) to transmit the pathogen, hence ϕ*→2* ϕ:
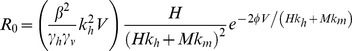
(27)


**Figure 7:**
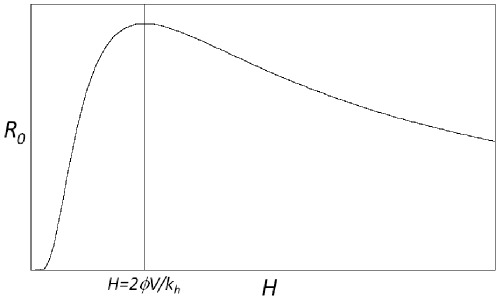
The response of *R_0_* to varying population of host animals (*H*) in a single host system, using the vector-interference model. Compare this response to the discontinuous model in Figure 2.

The functional form of the response of *R_0_* to *H* in the single host model is shown in Figure 7; the discontinuity at 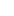 seen in Figure 2 has been replaced by a smooth transition from saturation to feeding site availability.

We find the locus of maximum *R_0_* as before (Equation 14) by taking the derivative with respect to *H*, and the positive solution is given by:
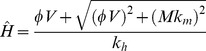
(28)


**Figure 8:**
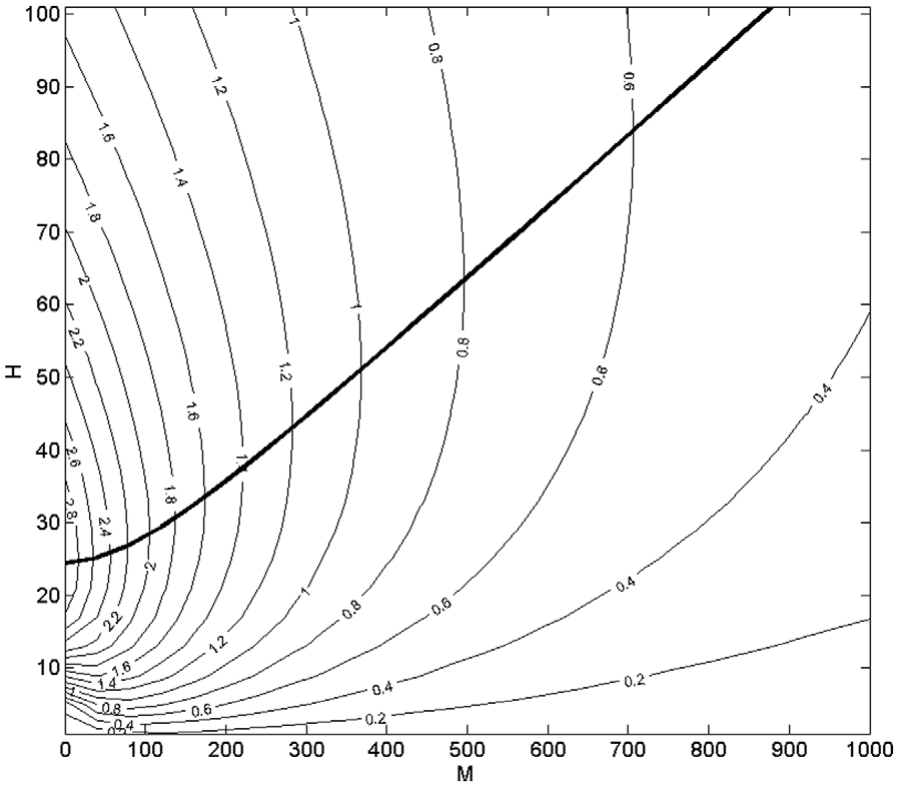
Predicted *R_0_* in the model with intraspecific feeding interference. Note that although there is no fully saturated region as there is in Figure 3, the locus of maximum *R_0_* (heavy line) does not pass through the origin.

The results are shown in Figure 8, which gives the contours of *R_0_* on axes of varying population sizes of competent (*H*) and incompetent (*M*) hosts. Comparing this with our original result for the one-host system, the peak of *R_0_* for *M = 0* occurs at a value of *H* lower by a factor of *2* ϕ than the value predicted by the discontinuous model. For 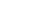:
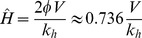
(29)


In *H-M* space, the vector interference model shows a similar form (Figure 8) to that of Figure 3, but without the saturated region at low *N* being demarcated by a discontinuity. The locus of maximum *R_0_* does not pass through the origin, despite the absence of a discontinuous saturated region.

## Discussion

We have examined a model showing dilution or amplification effects when the number of feeding sites on the host animals is limited. Our analysis shows how the presence of additional, reservoir-incompetent, host animals can affect the basic reproduction ratio *R_0_* both by dilution and amplification. Surprisingly, *R_0_* varies non-monotonically under a wide range of conditions. First, we considered a system with only a single host, and no incompetent alternatives for the vectors to feed upon. In this system, *R_0_* peaks at the boundary between saturated feeding sites (more vectors than sites) and excess feeding sites (more sites than vectors). This has important and counter-intuitive applications for the popular, but not always successful, pest control methods of reducing the number of disease host animals (e.g. [35]). This strategy can reduce *R_0_* only if the initial host population is below this boundary level. However, if the number of host animals is higher than this boundary level, reducing their numbers is likely to increase the risk of disease invasion and outbreak. While this result may at first seem counterintuitive, the explanation is straightforward; at high host population levels, vector loads are small, and so an infected individual will pass on the infection only to a small number of vectors. If feeding sites are saturated, the same infected individual will pass the infection to the maximum possible number of vectors each feeding session. This difference in the number of vectors infected by a host will be translated directly into a change in *R_0_*.

If such a non-monotonic response of *R_0_* to host numbers exists, it will be a significant challenge to address such an issue in the field. If it is impractical to estimate 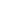 accurately, it is not possible to know whether reducing host numbers is an effective strategy for disease control. An indication of whether or not 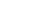 may be obtained in certain circumstances by estimating the occupancy of feeding sites on hosts; if the feeding sites on observed host animals are full, it may be reasonable to assume that *V<Hk_h_*, or *V<Hk_h_/2* ϕ and therefore that 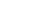


Turning to a two-host system, we show that *M-H* parameter space is divided into the same two regions of saturated and unsaturated feeding sites. Within the saturated region, *R_0_* is independent of the number of incompetent hosts and neither dilution nor amplification would be expected. The locus of the maximum *R_0_* tends to the origin, but is deflected to higher *H* values by the saturated region, confirming that if feeding sites were not limiting, we would observe a monotonic response of *R_0_* in the one host system. However, in the two host system, the non-monotonic response of *R_0_* is observed also in regions of parameter space far from the saturated region (Figure 3). This has two implications. Firstly, similar to the one host system, reducing the number of competent hosts will under certain circumstances increase *R_0_*. Secondly, altering the number of incompetent hosts could cross the maximum *R_0_* locus and cause a reversal of the effect of reducing host numbers. In other words, if at high levels of incompetent hosts (*M*), reducing the population of competent hosts (*H*) is an effective control strategy, at lower levels of incompetents, reducing competent hosts may increase disease prevalence.

These two opposite results of reducing *H* exist because the slope of the *R_0_* contours with respect to *M* can be either positive or negative (the contours “turn back” towards the origin for small *H*). This effect is seen also in other models of disease dynamics without the assumption of limited feeding sites. For instance, the model of [1] predicts *R_0_* from equations for the population dynamics of host and vector species. The *R_0_* isoclines he derives for the density-dependent transmission model show a concave response of *R_0_(H)*, but this too is modified by the presence of a second species. The implications of this are that other systems also may show this reversal of the response to control efforts.

Our model is specific in the consideration of the limiting nature of vector feeding sites. Previous works have concentrated on population dynamic effects (e.g. [36]), and in fact specifically exclude them to show that both dilution and amplification can exist without dynamic changes in population sizes – we compare only stable populations with a different ratio of competent to incompetent hosts. A further simplification of our model is that we assume perfect vector searching for feeding sites, although our vector interference model introduces an element of probability of feeding failure. In addition, host grooming may in practice produce quite different results from the case where feeding sites are physically limited, as partial blood meals may still be sufficient for disease transmission. Like many mathematical models in ecology, quantitative results are dependent on accurate estimation of model parameters. However, we take a different approach, demonstrating the qualitative and general characteristics of such a system [37]. Despite this, our parameter estimates, appropriate for a system involving a medium sized competent host and a smaller incompetent one, indicate that the observed non-monotonic behaviour of *R_0_* is likely to be present in real world systems.

The role of the dilution effect in disease systems has been the subject of some controversy. Keesing et al [38] reviewed the various mechanisms by which biodiversity could affect disease prevalence both positively and negatively. It is clear from their analysis is that no one treatment of all multihost disease systems can determine what the effect of increased biodiversity will be. Our model gives a specific demonstration of such a conclusion, since the particular biting regime that we describe does not generate results consistent with more general syntheses of multihost systems (e.g. [1]). Using the terminology of [38], our model shows a form of “encounter reduction”, i.e. reduced biting with increased numbers, although the effect is strongly non-linear.

Other authors have examined the effect of incompetent hosts on a vector-transmitted disease. Dobson [1] derived expressions for *R_0_* in a general system of multiple species capable of inter- and intra-specific infection. He concluded that in a density-dependent case, host diversity will always lead to increased values of *R_0_*, but frequency-dependent transmission will yield contours of *R_0_* similar to those that we have observed. Dobson [1] also derived expressions for the force of infection, and argued that control efforts should be directed against the species for which this expression is significantly larger. In our model, the incompetent host has a force of infection of zero but despite that, the presence of this species can determine whether or not controlling the competent host is an effective strategy. Gilbert et al [39] examined models of louping ill virus transmission in a three species system (grouse-hare-deer) with tick borne transmission. Their system emphasised the effect of host numbers (particularly deer) on tick populations, and they conclude that virus prevalence will increase with increasing deer numbers, but drop as the number of deer continue to increase and the dilution effect becomes dominant. The modelling of the rescue effect observed by adding deer to a hare-grouse system incapable of maintaining the virus could benefit from an examination of feeding site saturation, since sites are likely to become saturated on the hare and grouse, but less likely to be limiting on the deer.

The results we have shown here demonstrate the importance of incorporating specific details of disease ecology into predictive models. Vector transmission is far from the approximation of mass action [40] and predictions made on the basis of more simplistic models may be misleading. In particular, we predict a potential detrimental effect of naïve host-control techniques at certain levels of host abundance. Specific predictions of when host-control will produce the desired reductions in disease risk, and validation of those predictions, will be major challenges.
